# Anticodon-edited tRNA enables translational readthrough of *COL4A5* premature termination codons

**DOI:** 10.1371/journal.pone.0330804

**Published:** 2025-12-19

**Authors:** Kohei Omachi, Joseph J. Porter, John D. Lueck, Jeffrey H. Miner

**Affiliations:** 1 Division of Nephrology, Department of Medicine, Washington University School of Medicine, St. Louis, Missouri, United States of America; 2 Laboratory for Tissue Microenvironment, RIKEN Center for Biosystems Dynamics Research (BDR), Kobe, Hyogo, Japan; 3 Department of Pharmacology and Physiology, University of Rochester School of Medicine and Dentistry, Rochester, New York, United States of America; 4 Department of Neurology, University of Rochester School of Medicine and Dentistry, Rochester, New York, United States of America; 5 Center for RNA Biology, University of Rochester School of Medicine and Dentistry, Rochester, New York, United States of America; Medical Faculty Mannheim, University of Heidelberg, GERMANY

## Abstract

Alport syndrome is caused by variants in *COL4A3*, *COL4A4*, or *COL4A5*, which encode the α3α4α5 chains of type IV collagen. These variants result in defects in the glomerular basement membrane (GBM) and impaired kidney function. Nonsense variants result in truncated proteins lacking the NC1 domain, thereby preventing proper GBM assembly and function and causing the most severe forms of the disease. Restoring full-length protein expression represents a potential therapeutic strategy for Alport syndrome and related disorders. Anticodon-edited transfer RNAs (ACE-tRNAs), which promote premature termination codon (PTC) readthrough, have shown promise in diseases such as cystic fibrosis, but their application in Alport syndrome remains unexplored. To assess the potential of ACE-tRNAs for PTC readthrough of *COL4A5* nonsense variants, we employed a C-terminal NanoLuc-fused COL4A5 reporter system in which luminescence is produced only upon full-length protein translation. We introduced ACE-tRNAs into HeLa and 293T cells expressing one of four *COL4A5* nonsense variants (*S36X, R1563X, S1632X, and R1683X*) identified in patients with X-linked Alport syndrome. Readthrough efficiency was evaluated via NanoLuc luminescence and western blotting. Furthermore, we assessed the efficiency of ACE-tRNA-restored α3α4α5 heterotrimer formation using a split NanoLuc-based assay. Our results show that application of ACE-tRNAs led to restored C-terminal luminescence across all four COL4A5 nonsense variants, indicating successful readthrough and full-length translation. Moreover, the restored COL4A5 proteins formed α3α4α5 heterotrimers. These findings support ACE-tRNA-mediated nonsense suppression as a promising therapeutic strategy for Alport syndrome, with the potential to restore GBM integrity in patients harboring nonsense variants.

## Introduction

Nonsense variants, which introduce premature termination codons (PTCs), result in the production of truncated proteins that are often nonfunctional and are therefore frequently associated with severe phenotypes across a wide range of genetic diseases, including cystic fibrosis [1] and Duchenne muscular dystrophy [2]. One promising therapeutic approach is PTC readthrough, in which the translational machinery bypasses the premature termination codon, allowing for the synthesis of full-length proteins. This phenomenon has been extensively investigated, particularly in the context of small molecules that can induce readthrough. Among these, aminoglycosides such as gentamicin have long been recognized as PTC readthrough inducers [3–5]. In recent years, derivatives of aminoglycosides with improved readthrough efficiency and reduced toxicity have been developed, offering potential clinical benefits [6,7]. Despite these advancements, achieving therapeutically meaningful levels of readthrough remains a major challenge. To address this limitation, combination approaches incorporating readthrough potentiators have emerged as a complementary strategy [8,9]. These compounds enhance the activity of primary readthrough agents and may enable lower, less toxic dosages while improving functional protein restoration.

Another emerging strategy to bypass premature termination codons involves the use of anticodon-edited transfer RNAs (ACE-tRNAs) [10–12]. These engineered tRNAs carry a modified anticodon that enables them to specifically recognize premature stop codons and insert the original amino acid that should be present in the wild-type protein, thereby restoring the synthesis of full-length, functional proteins. Compared to aminoglycosides and their derivatives, ACE-tRNAs exhibit significantly higher readthrough efficiency [13]. Aminoglycoside-induced readthrough depends on near-cognate tRNAs, which often insert amino acids that differ from the original [14], and are active at both sense and nonsense codons [15,16]; thus, the resulting proteins may contain substitutions that impair function or stability. In contrast, ACE-tRNAs offer greater fidelity by directing the insertion of the correct amino acid, enhancing the likelihood of producing fully functional proteins.

Alport syndrome is the second most common genetic kidney disease. It is characterized by a glomerular filtration defect followed by progressive chronic kidney disease that eventually leads to kidney failure [17,18]. Alport syndrome is caused by loss-of-function variants in one of the genes encoding the type IV collagen α3α4α5 network—*COL4A3* [19,20], *COL4A4* [21], or *COL4A5* [22]. Among the many variants found in these genes, nonsense variants represent approximately 6% of cases. While missense variants are more common, nonsense variants are typically associated with a more severe clinical phenotype due to their impact on protein truncation and loss of function.

The type IV collagen α3α4α5 is synthesized as a heterotrimer, initiated through interactions at the C-terminal non-collagenous (NC1) domain, followed by stabilization via the collagenous domain and the N-terminal 7S domain [18]. In the case of nonsense variants, the resulting premature termination codon leads to truncated collagen chains that lack the NC1 domain, making them incapable of trimerization and thus unable to form a functional GBM scaffold. Given this pathogenic mechanism, therapeutic strategies aimed at restoring full-length type IV collagen proteins provide a rational and potentially disease-modifying treatment avenue for individuals with nonsense variant-associated Alport syndrome. Although ACE-tRNA therapy has been explored in various diseases using cultured cells [10,13,23–28] and mouse models [29,30], its potential application in Alport syndrome has not yet been investigated.

In this study, we evaluated the therapeutic potential of ACE-tRNAs to promote readthrough of PTCs in *COL4A5*. Utilizing a NanoLuc-based translational reporter system, we demonstrated that ACE-tRNAs effectively induce full-length COL4A5 protein expression in cultured cells expressing nonsense mutation-containing cDNAs. Importantly, the restored full-length COL4A5 protein was capable of forming heterotrimeric complexes with COL4A3 and COL4A4, suggesting that the translation products are not only structurally complete but also functionally competent. These findings establish a proof-of-concept for ACE-tRNA-mediated therapy in nonsense variant-driven Alport syndrome, supporting further development toward in vivo therapeutic applications.

## Materials and methods

### Cell culture

HeLa cells (ATCC, #CCL-2) were purchased from ATCC and maintained in Minimum Essential Medium (Gibco, # 11095080) supplemented with 10% FBS (Gibco, # A5670701), 1% penicillin streptomycin (Gibco, # 15140122) at 37°C in a humidified 5% CO_2_ incubator. 293T cells were purchased from ATCC and maintained in Dulbecco’s Modified Eagle Medium (Gibco, # 10644633) supplemented with 10% FBS (Gibco, # A5670701), 1% penicillin streptomycin (Gibco, # 15140122) at 37°C in a humidified 5% CO_2_ incubator. One day before transfection, cells were trypsinized and seeded into 6-well tissue culture plates (TPP, #92006). At 70–80% confluency, cells were transfected using Xfect transfection reagent (Takara, #631317) for HeLa and FuGENE 6 transfection reagent (Promega, # E2691) for 293T cells according to the manufacturer’s instructions. One day after transfection, the cells were trypsinized and seeded into Nunc 96-well white-bottom plates (Thermo Scientific, # 136102). Two days after transfection, cells were used for luciferase assays. Luciferase assays were conducted using DMEM without phenol red (Gibco, #11054020). All experiments used media containing 1% penicillin-streptomycin, as it does not impact PTC readthrough (S1 Fig).

For nonsense-mediated mRNA decay (NMD) inhibition experiments, cells were treated with 0.5 µM CC-90009 (Selleck, #S9832) for 24 hours. CC-90009 is a cereblon E3 ubiquitin ligase inhibitor that blocks NMD by knocking down eukaryotic release factor 3a (eRF3a), a key mediator of translational termination [8,31].

Primary kidney cells were isolated as previously described [32]. Briefly, the kidneys were removed from anesthetized mice and perfused with PBS. The kidneys were minced into 1–2 mm² pieces. Small pieces of tissue were incubated in 1 mg/mL collagenase and 100 U/mL DNase I for 30 min at 37°C. The digested tissues were filtered through a 100 µm cell strainer and centrifuged at 200 g for 5 min. After removing the supernatant, the cell pellet was resuspended in culture medium (RPMI-1640, Gibco # 11875093, supplemented with 10% FBS, 1% penicillin-streptomycin). Cells were cultured on a type I collagen-coated dish. During the first passaging, cells were dissociated by 0.25% trypsin/EDTA, filtered through a 30 µm cell strainer to remove debris, replated on type I collagen-coated dishes, and then used for the experiments.

### Plasmid DNA

Plasmids for the NanoLuc-based COL4A5 translational reporter assay were generated as previously described [32]. To normalize for transfection efficiency, the pGL4.54 [luc2/TK] vector (Promega, # E5061) was co-transfected as an internal control. The ACE-tRNA expression constructs—pUC57-4xACE-tRNA^Ser^_UGA_ and pUC57-4xACE-tRNA^Arg^_UGA_—used in this study were previously reported [10]. For all transfection experiments, except those assessing the effect of ACE-tRNA gene dosage, ACE-tRNA constructs were co-transfected at one-tenth the total mass of the *COL4A5*-NLuc reporter plasmid (mass ratio 1:0.1; equivalent molar ratio 1:0.29).

For the split-NanoLuc-based heterotrimer assay, NanoBiT® Large BiT (LgBiT)-tagged nonsense mutant *COL4A5* constructs were created by site-directed mutagenesis using the pFC34K LgBiT TK-Neo Human COL4A5: WT plasmid as a template [33]. Mutagenesis was performed with the following primers: S36X_forward: ctatgggtgttctccaggatGaaagtgtgactgcagtggc, S36X_reverse: gccactgcagtcacactttCatcctggagaacacccatag, S1632X_forward: cctgcttggaagagtttcgttGagctcccttcatcgaatg, S1632X_reverse: cattcgatgaagggagctCaacgaaactcttccaagcagg, R1563X_forward: gcatccagccattcattagtTgatgtgcagtatgtgaagctcc, R1563X_reverse: ggagcttcacatactgcacatcAactaatgaatggctggatgc, R1683X_forward: ggacacgaattagcTgatgtcaagtgtgcatg, R1683X_reverse: catgcacacttgacatcAgctaattcgtgtcc.

To generate NMD-sensitive COL4A5 translational reporter constructs, the *Luc2* gene in the pKC-4.04 vector (Addgene, # 112085) [34] was replaced with the *COL4A5-Nluc* gene. Both the pKC-4.04 backbone and the *COL4A5-Nluc* insert were amplified using PrimeSTAR Max DNA polymerase (Takara, # R045A), with the following primers: pKC-4.04_forward: gcgaattcatcgatagatctgatatc, pKC-4.04_reverse: ggttaattctgacggttcactaaac, COL4A5-Nluc_forward: gtgaaccgtcagaattaaccATGAAACTGCGTGGAGTCAGCCTGG, COL4A5-Nluc_reverse: agatctatcgatgaattcgccgccagaatgcgttcgcacagccgc.

PCR amplicons were purified using the QIAquick PCR purification kit (Qiagen, # 28104) and then assembled using NEBuilder HiFi DNA Assembly Master Mix (NEB, # E2621S). The integrity of the final constructs was confirmed by Sanger sequencing. Splicing of the HBB intron in the NMD-sensitive constructs was assessed by RT-PCR.

### RNA extraction and quantitative RT-PCR analysis

RNA was isolated from transfected cells using TRIzol reagent (Invitrogen, # 15596018) following the manufacturer’s instructions. Briefly, culture supernatants were removed, and cells were washed once with PBS before lysis in TRIzol. One-fifth volume of chloroform was added to the lysate, and the samples were centrifuged at 12,000 × *g* for 10 minutes. The aqueous phase was collected, followed by the addition of an equal volume of chloroform. After a second centrifugation (12,000 × *g*, 10 minutes), the aqueous phase was again collected. RNA was precipitated by adding an equal volume of isopropanol, followed by two washes with 70% ethanol. The RNA pellet was dissolved in TE buffer. RNA concentration and purity were determined using a NanoDrop 2000c spectrophotometer (Thermo Scientific). Purified RNA was reverse transcribed into cDNA using PrimeScript RT Master Mix (Takara, # RR036A) and used for PCR analysis with the following primers: pKC404 HBB FW1: ggctcacctggacaacctc, pKC404 HBB RV1: ccagcacaatcacgatcatattgc. The expected amplicon size was 773 bp for the unspliced transcript and 119 bp for the spliced transcript. *COL4A5-Nluc-HBB* mRNA expression was analyzed by quantitative reverse transcription PCR using FastSYBR Green Master Mix (Applied Biosystems, # 4385612) on a QuantStudio 6 Flex Real-Time PCR System (Applied Biosystems), following the manufacturer’s instructions.

### Luciferase assays

Both NanoLuc- and split–NanoLuc–based luciferase assays using the Nano-Glo® Dual-Luciferase® Reporter (NanoDLR™) Assay were performed as previously described [32]. Briefly, for the NanoLuc readthrough assay, supernatants were removed from cells cultured in 96-well white-bottom plates that had been transfected in quadruplicate. Cells were then lysed with firefly luciferase substrate buffer, incubated for 10 minutes at room temperature, and luminescence was measured. Subsequently, NanoLuc luciferase substrate buffer was added to each well, followed by a 10-minute incubation at room temperature, and luminescence was again measured. For the split–NanoLuc–based heterotrimer formation assay, both supernatants and cells were used, and luminescence was measured in the same manner as described for the NanoLuc luciferase assay. NanoLuc luminescence was normalized to firefly luminescence. Luminescence was measured using the GloMax-Multi Detection System (Promega).

### Statistics

Statistical parameters are provided in the figure legends. Luciferase assays were conducted in quadruplicate cell cultures. Statistical significance was assessed using Student’s t-test. Differences with P values less than 0.05 were considered statistically significant.

## Results

### ACE-tRNAs induce full-length protein expression in nonsense mutant *COL4A5* cDNA-expressing cells

**Fig 1:**
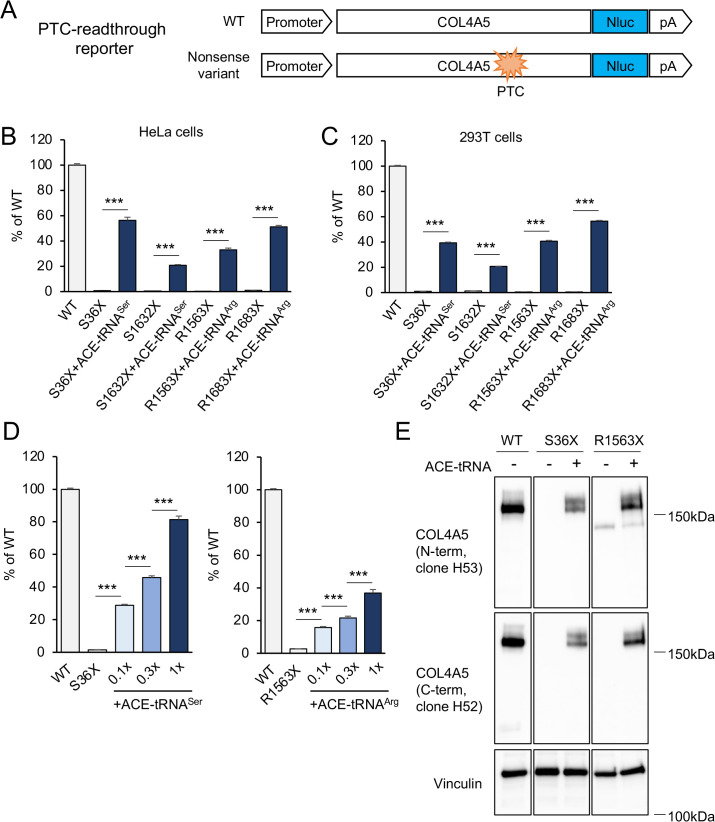
ACE-tRNA-mediated *COL4A5* PTC readthrough. **(A)** NLuc-based COL4A5 translation reporter constructs. The wild-type *COL4A5-NLuc* construct encodes a full-length COL4A5 protein fused to a C-terminal NLuc tag. Constructs harboring a PTC within the COL4A5 coding sequence led to truncated translation products that lack the C-terminal NLuc tag. **(B, C)** Luminescence was measured in lysates from HeLa cells or 293T cells co-transfected with *COL4A5-NLuc* (WT, S36X, S1632X, R1563X, or R1683X), HSV-*TK-Luc2*, and either ACE-tRNA^Ser^ or ACE-tRNA^Arg^ constructs. ACE-tRNA treatment promoted translational PTC readthrough in both HeLa and 293T cells, restoring expression of full-length COL4A5 protein. **(D)** Dose-dependent ACE-tRNA treatment induced readthrough of both *COL4A5-S36X* and *COL4A5-R1563X* in HeLa cells, with luminescence levels increasing in proportion to the amount of ACE-tRNA plasmid transfected. The ACE-tRNA expression construct was transfected at 0.1 × , 0.3 × , and 1× of the original plasmid amount (mass ratios 1:0.01, 1:0.03, and 1:0.1, relative to *COL4A5-NLuc*), corresponding to the bars shown from left to right in the graph labeled “+ACE-tRNA”. **(E)** The size of ACE-tRNA-induced full-length COL4A5 protein in HeLa cells was assessed by Western blotting using monoclonal rat IgG anti-COL4A5 (clone H53 for N-term and clone H52 for C-term). The rescued COL4A5 proteins displayed the expected molecular weight, confirming full-length translation. Vinculin was used as a loading control. Error bars indicate the mean ± SE (n = 4). Statistical analysis was performed using Student’s t-test. ***, P < 0.005 vs. non-rescued nonsense mutant.

To assess the full-length translation of *COL4A5* mRNA, a previously developed NanoLuc- (NLuc-) based reporter system was used [32]. The wild-type *COL4A5-NLuc* construct encodes a full-length COL4A5 protein fused to a C-terminal NLuc tag, resulting in detectable luminescence upon translation. In contrast, constructs harboring a premature termination codon (PTC) within the *COL4A5* coding sequence (S36X (UGA), S1632X (UGA), R1563X (UGA), R1683X (UGA)) lead to truncated translation products that lack the C-terminal NLuc domain, thereby abolishing luminescence (Fig 1A). This system allows for sensitive monitoring of translation efficiency and the effects of nonsense variants.

In both HeLa and 293T cells, luminescence was greatly reduced in the four nonsense mutant constructs compared to the wild-type, consistent with premature termination of translation. However, co-expression with ACE-tRNAs resulted in a substantial increase in luminescence across all four mutants (Fig 1B and 1C), indicating restoration of translation through PTC readthrough. The level of luminescence recovery ranged from ~20% to ~55%, exceeding that induced by high-dose G418, a conventional readthrough-inducing antibiotic [32]. This readthrough activity was dependent on the ACE-tRNA gene dose (Fig 1D). Furthermore, Western blot analysis using both N-terminal and C-terminal anti-COL4A5 antibodies confirmed the presence of full-length COL4A5 protein in ACE-tRNA-treated samples, but not in untreated nonsense mutants (Fig 1E). These results collectively demonstrate that ACE-tRNA effectively facilitates PTC readthrough and restores translation of full-length COL4A5 protein.

### ACE-tRNAs stabilize nonsense mutant *COL4A5* mRNA less effectively than an NMD inhibitor but promote PTC readthrough more robustly

**Fig 2:**
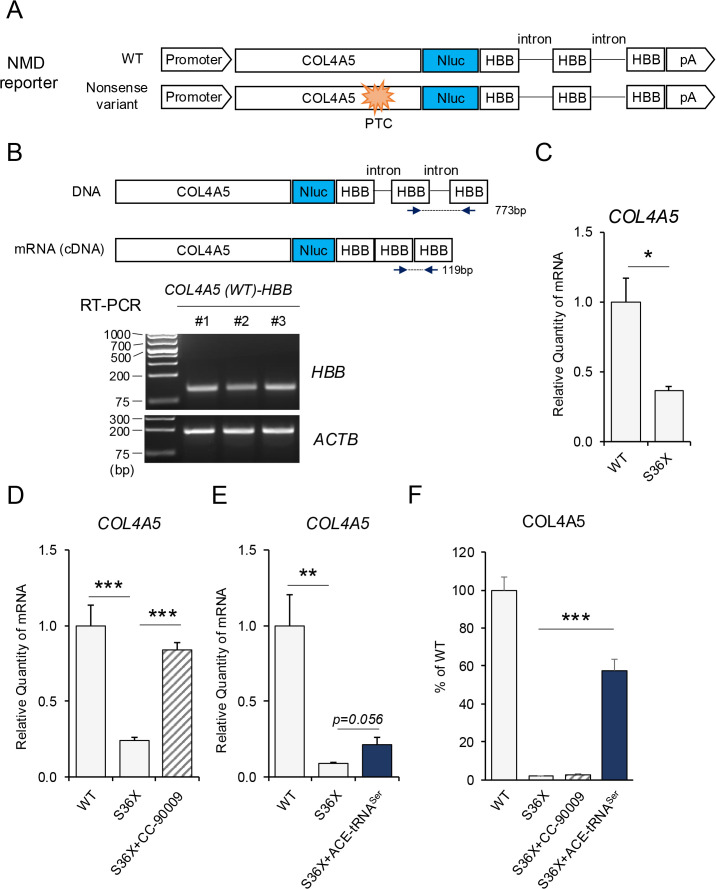
ACE-tRNA stabilizes nonsense-mutant *COL4A5* mRNA. **A)** Schematic of nonsense-mediated decay (NMD)-sensitive *COL4A5-NLuc* translation reporter constructs containing introns derived from the human β-globin (*HBB*) gene. **(B)** RT-PCR confirmed proper splicing of the *COL4A5-NLuc-HBB* exon-intron reporter construct in HeLa cells. **(C)** qRT-PCR shows reduced mRNA levels in cells expressing the *COL4A5-S36X* nonsense variant, consistent with NMD targeting in HeLa cells. **(D, E)** Treatment with the NMD inhibitor CC-90009 or expression of ACE-tRNA^Ser^ (10% mass ratio relative to *COL4A5-Nluc*) partially restored *COL4A5-S36X* mRNA levels, indicating stabilization via NMD inhibition in HeLa cells. **(F)** PTC readthrough was assessed by measuring NLuc expression following treatment with an NMD inhibitor or ACE-tRNA expression in HeLa cells. ACE-tRNA induced PTC readthrough significantly more effectively than NMD inhibition alone, highlighting its dual role in suppressing translation termination and partially rescuing transcript stability. Error bars indicate the mean ± SE (n = 4). Statistical analysis was performed using Student’s t-test. ***, P < 0.005, **, P < 0.001, *, P < 0.05 vs. non-rescued nonsense mutant.

Nonsense variants not only prematurely terminate translation but also reduce mRNA levels through nonsense-mediated decay (NMD). This process is regulated by the exon junction complex, which determines whether a transcript is subject to NMD [35]. Consequently, cDNA constructs containing PTCs but lacking introns are typically resistant to NMD. However, some introns in *COL4A5* are exceptionally large (e.g., intron 2 is 19,258 bp), making it technically challenging to incorporate native introns into expression constructs. To address this limitation, we employed Hemoglobin Subunit Beta (HBB) exons and introns, which have been successfully used to generate nonsense-mutated cDNAs sensitive to NMD [24,34,36]. Accordingly, to examine the effect of ACE-tRNA on mRNA stabilization, NMD-sensitive *COL4A5-NLuc* constructs were generated by incorporating both *HBB* exons and introns (Fig 2A). To confirm correct splicing, mRNA was isolated from cells expressing the *COL4A5-NLuc-HBB* construct and analyzed by RT-PCR using primers spanning two distinct *HBB* exons. The RT-PCR results demonstrated successful splicing, as the *HBB* intron was removed (Fig 2B). Introduction of the S36X nonsense mutation led to a reduction in *COL4A5-NLuc-HBB* mRNA levels (Fig 2C), which was rescued by treatment with the potent NMD inhibitor CC-90009 [31] (Fig 2D). These data show that the mutant *COL4A5-NLuc-HBB* constructs were indeed subject to NMD. Co-expression of ACE-tRNA modestly increased *S36X COL4A5-NLuc-HBB* mRNA levels, but this effect was weaker than that of the NMD inhibitor CC-90009 (Fig 2E). However, the efficiency of PTC readthrough, as indicated by NLuc induction, was substantially higher with ACE-tRNA expression compared to CC-90009 treatment (Fig 2F). While ACE-tRNAs strongly stabilize mRNA in other nonsense mutation contexts [28–30], the degree of mRNA stabilization observed in nonsense *COL4A5* overexpressing cells was weaker than expected. Nevertheless, robust PTC readthrough was consistently observed. Together, these findings support previous studies that ACE-tRNAs exert a dual role in suppressing nonsense mutations by promoting both PTC readthrough and mRNA stabilization [13].

### The full-length COL4A5 protein produced via ACE-tRNA-mediated readthrough forms heterotrimers

**Fig 3:**
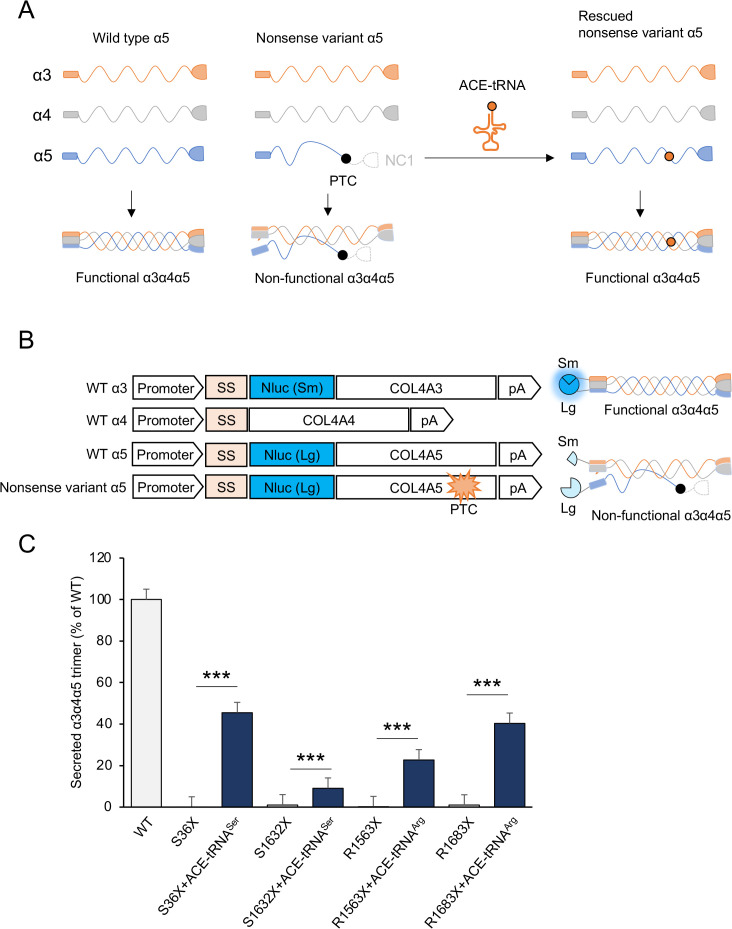
COL4-α3α4α5 heterotrimer formation in ACE-tRNA-mediated PTC readthrough products. **(A)** Schematic of the split-NLuc-based COL4A3/4/5 heterotrimer assembly reporter system. The split-NLuc fragments (SmBiT and LgBiT) were fused to COL4A3 and COL4A5, respectively; they generate luminescence only upon proper assembly of the α3α4α5 heterotrimer. Nonsense variant COL4A5 lacks the C-terminal NC1 domain and is unable to form a heterotrimer. **(B)** Luminescence was measured in culture supernatants from HeLa cells co-transfected with *COL4A3*-SmBiT, *COL4A4*, *COL4A5*-LgBiT, HSV-*TK-Luc2*, and ACE-tRNA constructs. ACE-tRNA constructs were co-transfected at one-tenth the total mass of the *COL4A5*-NLuc reporter plasmid. **(C)** ACE-tRNA expression restored COL4A5 translation in HeLa cells, enabling proper α3α4α5 heterotrimer formation, as evidenced by increased luminescence. These results confirm that ACE-tRNA-mediated readthrough not only restores full-length protein but also supports functional trimer assembly. Error bars indicate the mean ± SE (n = 4). Statistical analysis was performed using Student’s t-test. ***, P < 0.005 vs. non-rescued nonsense mutant.

Some of the readthrough products of nonsense variants in *COL4A5* mRNA induced by aminoglycosides were non-functional due to the suppression of premature termination by near-cognate tRNAs that insert an incorrect amino acid at the site of the PTC [37]. Since pathogenic missense variants in COL4A5 are known to impair formation of the α3α4α5 heterotrimers, accurate amino acid incorporation at the PTC site is critical. Thus, restoration of the wild-type residue during PTC readthrough can be as essential as the production of full-length protein for functional rescue. To evaluate the ability of readthrough products induced by ACE-tRNAs to form trimers, we employed a split-NLuc-based type IV collagen α3α4α5 heterotrimerization assay that has been previously described (Fig 3A and 3B) [33]. Nonsense mutant COL4A5 constructs showed no luminescence in the split-NLuc heterotrimer assay, indicating a lack of functional trimer formation. In contrast, treatment with the appropriate ACE-tRNAs restored luminescence in these mutants (Fig 3C), with induction levels ranging from ~9% to ~45% of wild-type. The relative efficacy across the four mutations mirrored the pattern observed for PTC readthrough efficiency in Fig 1B and 1C. These results suggest that ACE-tRNA not only promotes readthrough of premature stop codons but also facilitates the production of full-length, functionally competent COL4A5 capable of participating in α3α4α5 heterotrimer formation.

### ACE-tRNA rescued mRNA levels in primary kidney cells isolated from *Col4a5* mutant mice

**Fig 4:**
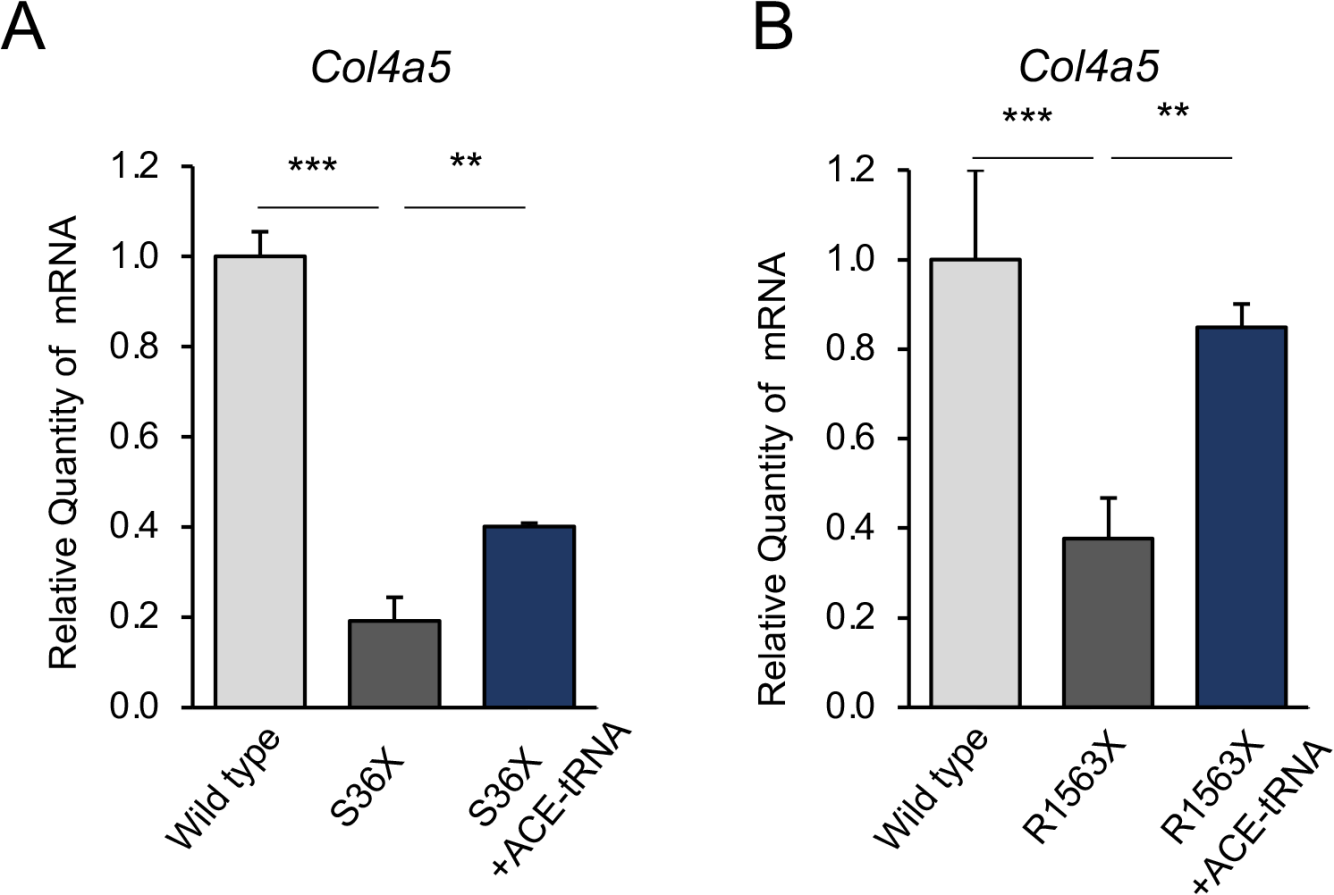
mRNA stabilization by ACE-tRNA in primary kidney cells isolated from *Col4a5* mutant mice. **(A)** qRT-PCR analysis showing reduced *Col4a5* mRNA levels in primary kidney cells expressing the endogenous *Col4a5-S36X* mutation, while ACE-tRNA expression for 2 days increased *Col4a5* mRNA levels in mutant cells. **(B)** qRT-PCR analysis showing reduced Col4a5 mRNA levels in primary kidney cells expressing the endogenous *Col4a5-R1563X* mutation, while ACE-tRNA expression for 2 days increased mRNA levels in mutant cells. Adenoviral infection was performed at a multiplicity of infection (MOI) of 100. Error bars indicate the mean  ±  SE (n= = 4). Statistical analysis was performed using Student’s t-test. ***, P  << 0.005, **, P  << 0.001 vs. non-rescued nonsense mutant.

Finally, we examined the effect of ACE-tRNA on the stabilization of endogenous *Col4a5* mRNA in primary kidney cells harboring nonsense mutations. In the kidney, both glomerular epithelial podocytes and tubular cells express *COL4A5*. We isolated bulk kidney cells from wild-type and two different *Col4a5* mutant mice (*Col4a5-*S36*X and -R1563X*) and introduced either scrambled tRNA or ACE-tRNA via adenoviral infection. Two days after infection, *Col4a5* mRNA levels were analyzed by qRT-PCR. Both S36X and R1563X nonsense-mutant cells showed markedly reduced *Col4a5* mRNA levels compared with wild-type cells (Fig 4A and 4B), indicating that the mutant transcripts underwent NMD as expected. ACE-tRNA expression increased *Col4a5* mRNA levels by approximately twofold (Fig 4A and 4B), demonstrating that ACE-tRNA stabilizes nonsense-mutant transcripts in primary kidney cells. These findings support the efficacy of ACE-tRNA in a physiologically relevant context beyond conventional cDNA-overexpressing cell lines and are consistent with previous studies on *CFTR* [13,28,30] and *IDUA* [29].

## Discussion

Alport syndrome caused by nonsense variants typically presents a more severe phenotype compared to non-truncating mutations, such as missense variants [38,39]. This is because type IV collagen, the protein affected in Alport syndrome, requires an intact C-terminal domain for proper heterotrimer assembly [40]. Nonsense mutations result in the production of truncated proteins that lack this critical domain, rendering the protein completely non-functional. Therefore, therapeutic strategies aimed at restoring full-length type IV collagen hold significant promise for treating Alport syndrome caused by nonsense variants. In the present study, we demonstrated that ACE-tRNAs effectively induce PTC readthrough, resulting in the restoration of full-length COL4A5 protein expression in cultured cells. Notably, the readthrough efficiency achieved with ACE-tRNA was significantly higher than that observed with aminoglycosides, which are known as classical PTC translational readthrough-inducing drugs (TRIDs) [29]. These findings establish a proof of concept for ACE-tRNA-mediated therapy as a broadly applicable approach for treating nonsense variant-associated Alport syndrome, including cases where conventional readthrough inducers are ineffective.

PTC readthrough activity in mammalian cells was initially discovered using aminoglycoside antibiotics, which were found to suppress premature termination codons and allow translation of full-length proteins [14]. Among these, G418 is recognized as a potent TRID [41] and is widely used as a gold standard in experimental models evaluating the efficacy of PTC suppression. However, despite its strong readthrough activity, G418 is associated with significant nephrotoxicity and ototoxicity, limiting its potential for clinical use [14]. In response to these limitations, gentamicin and other novel classes of PTC readthrough compounds have been developed [42]. As a result, PTC readthrough therapy has emerged as a realistic and promising therapeutic option for genetic disorders caused by nonsense variants. Despite these advances, the efficacy of current PTC TRIDs remains suboptimal and is generally insufficient to achieve a complete cure.

ACE-tRNAs represent a distinct therapeutic modality that utilizes tRNA rather than small molecules to induce PTC readthrough. Our findings indicate that ACE-tRNAs mediate significantly greater PTC readthrough efficiency (~60%) than G418, as demonstrated when comparing the results shown here to those in our previous study conducted under the same experimental conditions (~30%) [32]. Although G418 has been widely used in experimental settings, it is not under clinical development due to toxicity. Therefore, to determine which modality provides superior readthrough efficiency, further careful comparisons with newer classes of aminoglycoside derivatives, with or without TIRD potentiators, will be necessary.

A key advantage of ACE-tRNAs is the ability to incorporate the correct amino acid at the site of the premature stop codon, so called “seamless” rescue of the PTC, ensuring that the resulting protein maintains wild-type sequence and function [10]. Using a split-NLuc collagen IV functional assay, we showed that ACE-tRNA treatment not only induced PTC readthrough, but also increased the levels of functional COL4A5 protein. This contrasts with traditional PTC TRIDs like aminoglycosides, which rely on near-cognate tRNA recruitment, often resulting in missense amino acid incorporation that can impair protein function [43]. This distinction is particularly important in the context of Alport syndrome, where many pathogenic missense variants—such as glycine substitutions within the collagenous Gly-X-Y repeats—disrupt the structure and function of type IV collagen [44]. In our previous work, we evaluated readthrough products induced by aminoglycosides and found that certain substitutions resulted in non-functional proteins [32], indicating that not all *COL4A5* nonsense variants are amenable to readthrough therapy with traditional agents. However, in most cases, some of the readthrough protein induced by aminoglycosides would be fully functional as either a wild-type protein or following the incorporation of another amino acid that allows proper COL4A5 function. Many laboratories are developing new types of TRIDs and potentiator compounds to increase drug potency [8,9], thereby increasing the amount of wild-type proteins and overcoming this issue.

In considering ACE-tRNAs as a platform therapeutic, it is also possible for many *COL4A5* nonsense mutations to be suppressed with one ACE-tRNA that incorporates an amino acid that supports a fully functional COL4A5 variant. This approach, akin to the use of TRIDS, would expand the Alport patient population to be treated with one therapeutic, thus reducing the burden of moving several ACE-tRNA sequences through clinical trials. This concept was recently demonstrated with functional rescue of the cystic fibrosis transmembrane conductance regulator (CFTR) channel harboring the four most common nonsense mutations (G542X, R553X, R1162X and W1282X) with a leucine encoding ACE-tRNA [28].

A potential consideration, however, is the off-target readthrough of native termination codons. This activity is substantially lower—approximately 100- to 1000-fold less—than that observed for PTC readthrough, as demonstrated by transcriptome-wide ribosome profiling of 3’ UTRs [10,26,29,30]. In the present study, we did not specifically evaluate this possibility. Because potential off-target readthrough may vary depending on the type of ACE-tRNA, further investigation using ribosome profiling or mass spectrometry-based approaches will be necessary.

Nonsense-mediated mRNA decay (NMD) plays a key role in the pre-translational regulation of nonsense variant protein synthesis [35]. Although there are exceptions, the majority of mRNAs containing a PTC are recognized and degraded by the NMD pathway, thereby suppressing the production of truncated proteins. Therefore, stabilization of PTC-containing mRNAs through NMD inhibition is regarded as an alternative and complementary approach to augment PTC readthrough efficiency. In this study, we adapted the well-characterized *HBB* splicing cassette [34] into our *COL4A5-NLuc* reporter system to investigate the impact of ACE-tRNA on NMD. The mRNA abundance of the *COL4A5-S36X-NLuc-HBB* construct was significantly reduced, consistent with active NMD, and was effectively rescued by the NMD inhibitor CC-90009 [31]. In contrast, the mRNA-stabilizing effect of ACE-tRNA on the same construct was modest and markedly weaker than that of the NMD inhibitor. Despite its modest impact on mRNA stabilization, ACE-tRNA treatment effectively induced full-length protein expression, whereas the NMD inhibitor did not. This observation is consistent with the mechanism of action of ACE-tRNA, which is designed to promote the incorporation of the correct amino acid at the PTC, thereby enabling the production of full-length functional proteins. However, other studies have clearly demonstrated that ACE-tRNA can stabilize mRNA expressed from a gene harboring a genomically encoded nonsense codon [13,28]. This discrepancy may be attributed to differences in the genes, cell types, or experimental systems used.

One limitation of the present study is the use of standard cell lines and an overexpression system, which may not fully recapitulate physiological conditions. Although we demonstrated mRNA stabilization by ACE-tRNAs in primary kidney cells derived from nonsense-mutant mouse models, it remains unclear how this stabilization enhances protein expression. This issue should be explored using primary podocytes, which are the main producers of the COL4A5 in the GBM, in a follow-up study. Importantly, our findings also showed that NMD inhibition alone did not lead to full-length protein expression, reinforcing the conclusion that potent PTC readthrough activity, rather than mRNA stabilization, is the critical factor for therapeutic efficacy.

In the context of clinical translation, small molecule-based TRIDs have a major advantage in systemic delivery. However, compared with conventional gene therapy, ACE-tRNAs also offer several notable advantages [11]. The compact expression cassette is small enough to be packaged into adeno-associated virus (AAV) vectors, which are widely used in gene therapy [29]. Additionally, because ACE-tRNAs can be delivered as an RNA-based therapeutic modality, they are also compatible with lipid nanoparticle (LNP) delivery systems [30], providing flexibility in therapeutic delivery platforms. Importantly, AAV-based therapies are known for their long-lasting effects, often requiring only a single administration, thereby minimizing the treatment burden. For example, in AAV-based gene therapy for muscular dystrophy, a single dose has demonstrated sustained therapeutic benefits over extended periods [45,46]. In the case of Alport syndrome, gene replacement therapy remains a challenge because the *COL4A5* cDNA is too large to be accommodated within AAV vectors. This limitation underscores the promise of ACE-tRNA as a long-acting therapeutic strategy that directly targets the underlying nonsense variants. By enabling the production of full-length, functional protein, ACE-tRNA represents an ideal approach for directly addressing the genetic defect in Alport syndrome due to nonsense variants.

In summary, the present study proposes the use of ACE-tRNAs as a promising therapeutic strategy for Alport syndrome caused by nonsense variants. ACE-tRNAs exhibit potent PTC readthrough activity and restore the original amino acid, enabling the production of full-length, functional proteins. Importantly, ACE-tRNAs can be customized to target all three types of nonsense codons, each with the appropriate amino acid, offering a precise and adaptable therapeutic platform. Our findings establish a proof of concept for ACE-tRNA–mediated therapy in Alport syndrome and provide a strong rationale for advancing to *in vivo* studies in the near future.

## Supporting information

S1 Fig ACE-tRNA-mediated COL4A5 PTC readthrough without penicillin/streptomycin. The effect of penicillin/streptomycin on basal readthrough and ACE-tRNA-induced full-length COL4A5 protein in transfected 293T cells was evaluated by Western blotting. Penicillin/streptomycin did not induce basal readthrough, as shown in lanes #2 and #3, compared with the penicillin/streptomycin-free condition (lanes #7 and #8). ACE-tRNA-mediated PTC readthrough was at a similar level in both conditions, with and without penicillin/streptomycin.
(PDF)

S2 Fig Uncropped membranes and gels. (PDF)
