# Supplementary material for: Anticodon-edited tRNA enables translational readthrough of COL4A5 premature termination codons
Source: PLoS One. 2025 Dec 19;20(12):e0330804. doi: 10.1371/journal.pone.0330804 (PMC12716684; doi:10.1371/journal.pone.0330804)
Supplement: S1 Fig — The effect of penicillin/streptomycin on basal readthrough and ACE-tRNA-induced full-length COL4A5 protein in transfected 293T cells was evaluated by Western blotting. Penicillin/streptomycin did not induce basal readthrough, as shown in lanes #2 and #3, compared with the penicillin/streptomycin-free condition (lanes #7 and #8). ACE-tRNA-mediated PTC readthrough was at a similar level in both conditions, with and without penicillin/streptomycin. (PDF) [file pone.0330804.s001.pdf]

Fig. S1

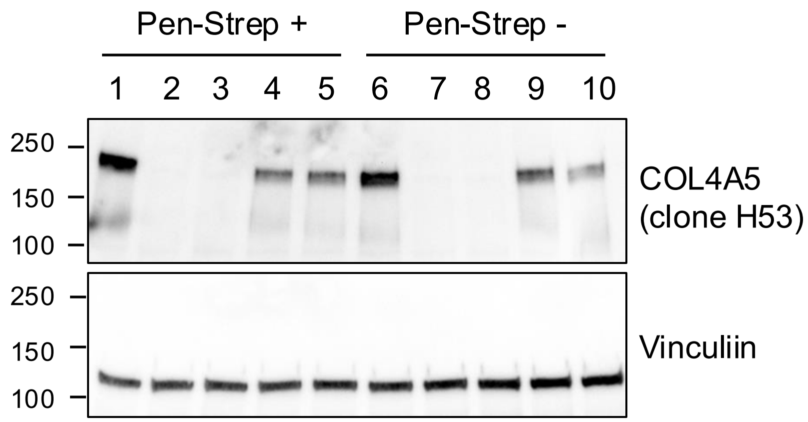

Pen-Strep +

1. WT COL4A5 + ACE-tRNA_NT
2. S36X-COL4A5 + ACE-tRNA_NT
3. S36X-COL4A5 + ACE-tRNA_NT
4. S36X-COL4A5 + ACE-tRNA_SerTGA
5. S36X-COL4A5 + ACE-tRNA_SerTGA

Pen-Strep -

6. WT COL4A5 + ACE-tRNA_NT
7. S36X-COL4A5 + ACE-tRNA_NT
8. S36X-COL4A5 + ACE-tRNA_NT
9. S36X-COL4A5 + ACE-tRNA_SerTGA
10. S36X-COL4A5 + ACE-tRNA_SerTGA

Fig. S1. ACE-tRNA-mediated COL4A5 PTC readthrough without penicillin/streptomycin condition

The effect of penicillin/streptomycin on basal readthrough and ACE-tRNA-induced full-length COL4A5 protein in transfected 293T cells was evaluated by Western blotting. Penicillin/streptomycin did not induce basal readthrough, as shown in lanes #2 and #3, compared with the penicillin/streptomycin-free condition (lanes #7 and #8). ACE-tRNA-mediated PTC readthrough was at a similar level in both conditions, with and without penicillin/streptomycin.
