# Supplementary figures and images for: Anticodon-edited tRNA enables translational readthrough of COL4A5 premature termination codons
Source: PLoS One. 2025 Dec 19;20(12):e0330804. doi: 10.1371/journal.pone.0330804 (PMC12716684; doi:10.1371/journal.pone.0330804)

**Fig. S2**

**Fig.1E**

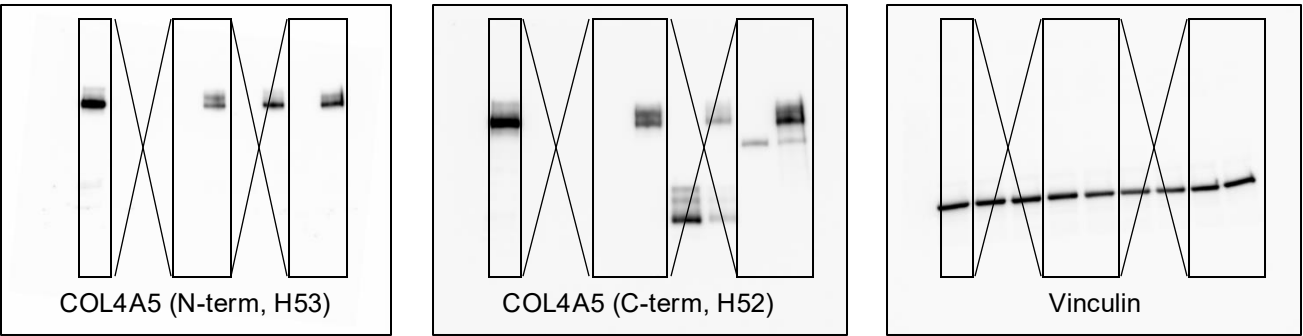

**Fig.2B**

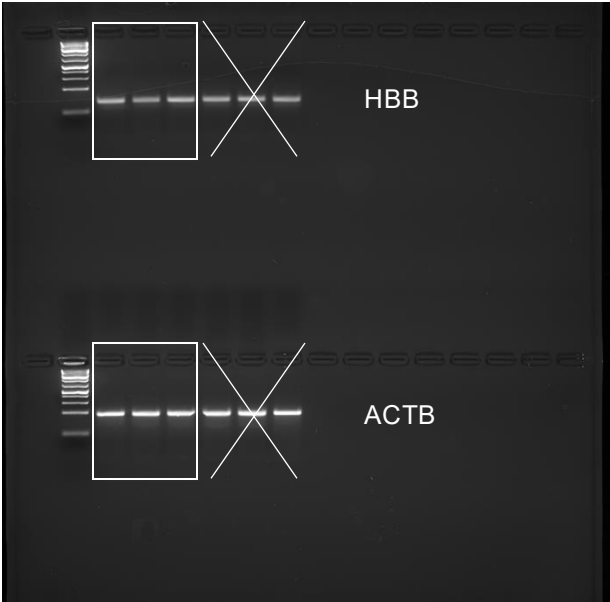

**Fig.S1**

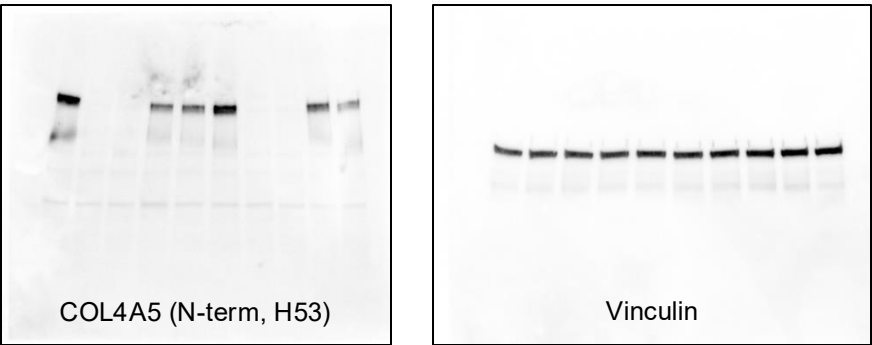

**Fig. S2. Uncropped membrane and gels**

Supplement: S2 Fig — (PDF) [file pone.0330804.s002.pdf]
